# Partial body cryotherapy exposure drives acute redistribution of circulating lymphocytes: preliminary findings

**DOI:** 10.1007/s00421-022-05058-3

**Published:** 2022-11-09

**Authors:** Catriona L. Rose, Helen McGuire, Kenneth Graham, Jason Siegler, Barbara Fazekas de St Groth, Corinne Caillaud, Kate M. Edwards

**Affiliations:** 1 Faculty of Medicine and Health, Sydney and School of Health Sciences, Discipline of Exercise and Sport Science, The University of Sydney, Charles Perkins Centre, Camperdown, Sydney, NSW 2006 Australia; 2 Faculty of Medicine and Health, Sydney and School of Medical Sciences, Discipline of Pathology and Ramaciotti Facility for Human Systems Biology, The University of Sydney, Sydney, NSW Australia; 3 Applied Research Programme, New South Wales Institute of Sport, Sydney, Australia; 4 Sport and Exercise Science, School of Science and Health, University of Western Sydney, Sydney, NSW Australia

**Keywords:** Partial body cryotherapy, Lymphocyte mobilisation, Acute stress response

## Abstract

Partial body cryotherapy (PBC) is proposed to alleviate symptoms of exercise-induced muscle damage (EIMD) by reducing associated inflammation. No studies have assessed acute PBC exposure on peripheral blood mononuclear cell mobilisation or compared these with cold water immersion (CWI), which may inform how PBC impacts inflammatory processes. This trial examined the impact of a single PBC exposure on circulating peripheral blood mononuclear cells compared to CWI or a control. 26 males were randomised into either PBC (3 min at − 110 to − 140 °C), CWI (3 min at 9 °C), or control (3 min at 24 °C), with blood samples, heart rate, and blood pressure taken before and after exposure. Cytometric analysis determined that CD8^+^ T-cell populations were significantly elevated after treatments, with PBC increasing CD8^+^ T cells to a greater degree than either CWI or CON. Natural killer cell counts were also elevated after PBC, with the increase attributed specifically to the CD56^lo^CD16^+^ cytotoxic subset. This provides the first evidence for the effect of PBC exposure on redistribution of immune cells. An increase in circulating leukocyte subsets such as CD8^+^ T cells and CD56^lo^CD16^+^ natural killer cells suggests that PBC may induce a transient mobilisation of lymphocytes. PBC may thus enable a more efficient trafficking of these cells from the circulation to the site of initial cellular insult from exercise, potentially accelerating the process of cellular recovery. This provides novel evidence on the use of PBC as a recovery treatment and may also have applicability in other clinical settings involving the recovery of damaged skeletal muscle.

## Introduction

The use of partial body cryotherapy (PBC) as a recovery treatment is premised on the theoretical basis that it will reduce inflammation leading to a decrease in the associated pain and loss of muscle function that accompanies exercise-induced muscle damage (EIMD) (Bleakley et al. 2012; Hohenauer et al. 2018). Cold water immersion (CWI) treatment involves exposures of between 9 and 11 °C; however, protocols for exposure to PBC expose participants standing in a chamber where the air temperature is cooled with nitrogen gas to − 110 °C or less. Proposed mechanisms for the effects of PBC include vasoconstriction and analgesia due to acute cold exposure as well as anti-inflammatory action (Hausswirth et al. 2011; Hohenauer et al. 2015). Compared to the effects of CWI, evidence for use of PBC to expedite recovery from muscle damage is unclear. Developing a clearer understanding of the physiological impact of PBC exposure, in comparison to CWI and a control group, may inform future applications of the novel treatment.

Responses to PBC have many similarities to those elicited by other types of stressors (psychological, physical, or chemical), in terms of an increase in heart rate, blood pressure, and a systemic increase in circulating catecholamines (Costello et al. 2012; Lombardi et al. 2017). One response that might be similarly expected, but has not been examined, is lymphocyte redistribution (Lubkowska et al. 2010; Poppendieck et al. 2013; Lombardi et al. 2017; Hohenauer et al. 2018). Recent studies have hypothesised that the acute cold shock response from PBC exposure may impact the distribution of immune cells involved in the inflammatory process, which could contribute to changes elicited by EIMD and potentially other immune responses (Poppendieck et al. 2013; Hohenauer et al. 2015).

Lymphocytosis, the increase of lymphocytes into the circulation, has commonly been observed in response to acute stress, regardless of the mode of stimulus. This effect is controlled primarily from stimulation of beta-adrenergic receptors by epinephrine, which increases in response to stress through sympatho-medullary axis activation (Benschop et al. 1996; Fragala et al. 2011). Acute stress-induced increases in circulating lymphocyte numbers are not uniform, and there is preferential mobilisation of specific immune cell subsets, including CD8 T cells and CD16^+^ NK cells, that exhibit tissue-homing or cytotoxic functionality (Campbell et al. 2009; Dhabhar et al. 2012; Campbell and Turner 2018). Differences in the magnitude of neuroendocrine-mediated immune cell mobilisation are generally associated with the mode and duration of the stressor (Fragala et al. 2011; Dhabhar 2014). After the initial stressor is removed, PBMC populations rapidly fall to below pre-stress levels, indicating that these cells have either trafficked to the target organ, to sites of immune activation, or back to the initial compartment (Viswanathan and Dhabhar 2005; Dhabhar et al. 2012). This process has been suggested to represent the activation of the immune system, preparing to defend from harmful antigens or play a role in the regeneration of injured tissue (Dhabhar 2009; Dhabhar et al. 2012; Campbell and Turner 2018).

Modifications in numbers and functionality of NK, B, and T cells have been well described using a variety of stressors. However, the acute effect of a single PBC treatment on cell mobilisation, particularly in contrast to CWI, is yet unknown. This trial aimed to investigate the effect of an acute PBC exposure on acutely redistributing circulating PBMC profiles in comparison to both cold water immersion (CWI) and a control (CON). These findings may inform the process of cellular recovery proposed with use of these cooling treatments. We hypothesised that an acute 3 min cold exposure would induce a stress response leading to increases in circulating PBMC subpopulations with cytotoxic and tissue-homing characteristics, with a greater response expected after PBC compared to CWI.

## Methods

The study was approved by both the Australian Institute of Sport Ethics Committee (approval number 20121211) and the University of Sydney Ethics Committee (approval number 2013/0430). Testing was conducted at the New South Wales Institute of Sport in Sydney in a randomised study design.

Male participants aged between 18 and 35 years were recruited by advertisement at the University of Sydney. Inclusion criteria included completion of the Physical Activity Readiness questionnaire (PAR-Q) and General Health Questionnaire (GHQ), and a screening for any recent illness or injury, or for the current use of antioxidant supplements, or anti-inflammatory or steroidal medication which may have impacted immune competence.

In total, 55 individuals were screened, 15 were excluded due to illness or injury, inability to commit to the trial requirements, or current use of anti-inflammatory medication. The 40 remaining participants provided informed consent and were randomly allocated a treatment (PBC *n* = 15, CWI *n* = 11 or CON *n* = 14), using a random numbers generator (randomization.com). BP and HR were collected and analysed for all 40 participants. Five participants failed to provide blood samples, and ten participants were excluded from analysis due to a low cell count from their initial sample. PBMC analysis was performed for 25 participants resulting in a final sample for PBMC analysis of PBC *n* = 8, CWI *n* = 9, and CON *n* = 9.

Participants were asked to refrain from physical activity and alcohol the day before testing and to abstain from caffeine and food intake 2 h prior to the testing session. Blood samples were taken prior to treatment exposure, and again, immediately after. At each of the collection time points, 9 mL of blood was collected into a heparin-coated vacutainer (BD Biosciences, San Jose, CA). Cell concentrations in the blood sample were determined using a Sysmex XP-300™ Automated Hematology Analyzer (Sysmex, Kobe, Japan).

HR and blood pressure were measured at the end of 15 min of seated rest and immediately after exposure. Heart rate (HR) was recorded using a wrist HR monitor (RS800CX Training Computer, Polar Electro, Kempele, Finland) and chest strap (H3 Heart Rate Sensor, Polar Electro, Kempele, Finland). Blood pressure was measured using an automatic self-inflatable cuff (HEM-7121 Automatic Blood Pressure Monitor, Omron, Kyoto, Japan).

During PBC treatment, participants stood upright with the head exposed in a single user cryocabin wearing gloves, soft boots, and shorts (Space Cabin Cryosauna Classic, Kherson, Ukraine) for 3 min between − 110 and − 140 °C following a safety protocol that has been referenced elsewhere (Fonda and Sarabon 2013; Ferreira-Junior et al. 2015). All safety requirements of exposure protocols were followed to prevent the risk of injury during exposure. The CWI treatment involved 3 min seated in water up to the xiphoid process in a specifically designed bath (iSprint bath, iCool, Gold Coast, Australia), with the water running through a chiller unit (iCool Lite Pump, Gold Coast, Australia) that maintained the temperature at 9 °C. Participants randomised to the CON treatment were instructed to sit quietly on a chair at room temperature of 24 °C for 3 min. A CWI immersion duration of 3 min was chosen to replicate the treatment exposure for PBC.

PBMCs were isolated from 9 mL of whole blood collected in heparin tubes according to previously published methods (Jaatinen and Laine 2007). Mass cytometry was used to precisely quantify the proportion and phenotype of immune cells circulating at the time of sample collection (Yao et al. 2014; Leipold and Maecker 2015). Cryopreserved PBMCs were thawed, assessed for viability, and stained for mass cytometric analysis according to previously published methods (McGuire et al. 2020). All antibodies were validated, pre-tittered, and supplied in per-test amounts by the Ramaciotti Facility for Human Systems Biology Mass Cytometry Reagent Bank. Reagent bank antibodies were purchased in a carrier-protein-free format and conjugated by the Ramaciotti Facility for Human Systems Biology with individual metal isotopes using the MaxPAR conjugation kit (Fluidigm, South San Francisco, CA) according to the manufacturer’s protocol. Data in FCS3 file format were collected using the Helios software (V6.3.119) and normalised across experiments using EQ Four Element Calibration Beads using data processing function within the acquisition software. FlowJo software was subsequently used to pre-gate on DNA^+^, live, CD45^+^ cells, and then to gate all cells into major immune cell populations. On average, more than 200,000 CD45 + cells were analysed per sample. Subsequent gating was performed to determine the variety of immune subsets assessed. This included T-cell subsets, B cells, monocytes, and NK cells, as detailed in Table 1 and published previously (McGuire et al. 2020). To factor in numeric changes in lymphocyte and monocyte populations, the size of immune subpopulations was scaled and expressed as cells per 10^9^/L, based on the cell counts measured on the automated full blood count analyser on the day of sample collection.

**Table 1:** PBMC populations

Cell type	Gating strategy	Time point	PBC (#/L blood)	CWI (#/L blood)	CON (#/L blood)
T cells × 10^9^/L blood	CD3 +	Rest	1.145 ± 0.331	0.997 ± 0.445	03771 ± 0.330
		Post	1.614 ± 0.689*^#^	1.147 ± 0.469	0.957 ± 0.329
CD4 + T cells × 10^9^/L blood	T cells > CD4 +	Rest	0.469 ± 0.216	0.536 ± 0.260	0.300 ± 0.153
		Post	0.539 ± 0.271	0.613 ± 0.279	0.484 ± 0.234*
CD8 + T cells × 10^9^/L blood	T cells > CD8 +	Rest	0.654 ± 0.184	0.461 ± 0.190	0.468 ± 0.238
		Post	1.045 ± 0.484*^#^	0.565 ± 0.208	0.474 ± 0.143
Tconventional CD4 + T cells × 10^9^/L blood	CD4 + T cells > CD127hi	Rest	0.427 ± 0.195	0.496 ± 0.248	0.3266 ± 0.145
		Post	0.492 ± 0.252*	0.568 ± 0.263*	0.443 ± 0.213*
Tregs × 10^9^/L blood	CD4 + T cells > CD25hi CD127lo	Rest	0.041 ± 0.024	0.040 ± 0.018	0.029 ± 0.013
		Post	0.046 ± 0.029	0.043 ± 0.022	0.040 ± 0.020
B cells × 10^9^/L blood	CD19 + CD20 + CD3-	Rest	0.257 ± 0.110	0.240 ± 0.082	0.169 ± 0.114
		Post	0.240 ± 0.149	0.237 ± 0.120	0.281 ± 0.152^#^
Dendritic cells (DCs) × 10^9^/L blood	CD3- CD19- CD56- HLADR + CD14-	Rest	0.131 ± 0.110	0.130 ± 0.052	0.261 ± 0.286
		Post	0.165 ± 0.167	0.135 ± 0.060	0.169 ± 0.211
mDCs × 10^9^/L blood	DCs > CD11c +	Rest	0.095 ± 0.102	0.080 ± 0.052	0.126 ± 0.155
		Post	0.114 ± 0.146	0.082 ± 0.066	0.094 ± 0.118
pDCs × 10^9^/L blood	DCs > CD123 +	Rest	0.012 ± 0.007	0.019 ± 0.015	0.021 ± 0.0312
		Post	0.023 ± 0.019*	0.022 ± 0.016	0.023 ± 0.041
NK cells × 10^9^/L blood	CD3- CD19- CD56 + HLADR-	Rest	0.218 ± 0.081*^#^	0.300 ± 0.167	0.262 ± 0.133
		Post	0.457 ± 0.212	0.394 ± 0.180	0.268 ± 0.167
CD16^−^ NK cells × 10^9^/L blood	NK cells > CD16-	Rest	0.099 ± 0.053	0.160 ± 0.088	0.168 ± 0.153
		Post	0.138 ± 0.072	0.181 ± 0.093	0.147 ± 0.178
CD16^+^ NK cells × 10^9^/L blood	NK cells > CD16 +	Rest	0.118 ± 0.078	0.133 ± 0.103	0.093 ± 0.052
		Post	0.315 ± 0.225*^#^	0.210 ± 0.108	0.120 ± 0.039
Classical monocytes × 10^9^/L blood	CD3- CD19- CD56- HLADR + CD14 +	Rest	0.401 ± 0.119	0.384 ± 0.128	0.464 ± 0.188^#^
		Post	0.481 ± 0.111	0.405 ± 0.095	0.395 ± 0.233
Non-classical monocytes × 10^9^/L blood	CD3- CD19- CD56- HLADR + CD14lo CD16 +	Rest	0.074 ± 0.049^#^	0.066 ± 0.048	0.131 ± 0.087^#^
		Post	0.119 ± 0.064	0.082 ± 0.045	0.130 ± 0.096

Observations of cell populations at 15 min of rest, and immediately post-exposure. ± Standard deviation, *denotes significant time effect of treatment group between rest and post-exposure, ^#^denotes a significant time-by-treatment effect where the treatment effect is significantly different to either, or both, other treatment groups (*p* ≤ 0.05). Cells were gated as T cells (CD3^+^, CD4^+^, CD8^+^), conventional (Tconventional) and regulatory T cells (Treg), B cells, dendritic cells, myeloid dendritic cells (mDCs), plasmacytoid dendritic cells (pDCs), natural killer (NK) cells (CD16^−^, CD16^+^), classical and non-classical monocytes. *PBC* partial body cryotherapy, *CWI* cold water immersion, *CON* control

Results from the rest and post time points between the three treatments were analysed using a two-way repeated-measures ANOVA (treatment × time) using SPSS software (IBM SPSS Statistics 24 Software, New York, USA), with a 95% confidence interval. All data are presented as mean ± standard deviation (SD). The Shapiro–Wilk test of normality was used to determine the distribution of data. Levene’s test of equality of error variances was used to verify the assumption that variances were equal between groups. The variance of means between groups at rest was analysed from results of the one-way repeated-measures ANOVA to ensure that there were no statistically significant differences in resting concentrations of variables between groups prior to treatment. The Bonferonni adjustment was used to reduce the risk of Type I error, and pairwise comparisons were used in cases that a significant effect was reported. Raw data were used, with outliers and missing data included in group analysis but not in any pairwise comparisons, and are reflected in the degrees of freedom reported.

## Results

Participant age was 23.4 (± 4.6) years, height was 179.2 (± 6.7) cm tall, and body mass was 78.5 (± 10.6) kg. Although randomisation was used, there was a significant difference in height between groups; participants in the CWI group were, on average, taller than PBC participants (*p* = 0.035), but not the CON group (*p* = 0.287). No significant differences were observed in either age or weight between groups.

At rest, HR was not significantly different between groups. However, significant treatment (*F* (3, 26) = 6.56, *p* ≤ 0.01, *ƞp*^2^ = 0.42) and time (*F* (1, 26 = 23.47, *p* ≤ 0.01, *ƞp*^2^ = 0.57) effects were recorded for HR. Immediately after exposure, the CON group increased by 10.3 (± 15) bpm, CWI group decreased by 3.3 (± 13.2) bpm, and the PBC group increased by 33 (± 27.4) bpm with the difference between PBC and CWI statistically significant (*p* = 0.04). There was no differential effect of treatments on systolic BP (*F* (4, 28) = 1.294, *p* = 0.296, *ƞp*^2^ = 0.16), but a significant increase with time post-treatment was present in all groups (PBC: 7.8 (± 13.4) mmHg, CWI: 14 (± 14.4) mmHg, CON: 3.5 (± 9.8) mmHg). Similarly, no significant time-by-treatment interaction was found in diastolic BP (*F* (4, 28) = 0.54, *p* = 0.76, *ƞp*^2^ = 0.07), while a significant time effect was found (*F* (2, 28) = 5.76, *p* = 0.01, *ƞp*^2^ = 0.30) with increases after exposure across all three groups [PBC: + 5.3 (± 16.3) mmHg, CWI: + 12.2 (± 14.1) mmHg, and CON: + 8.14 (± 16.3) mmHg].

**Fig. 1:**
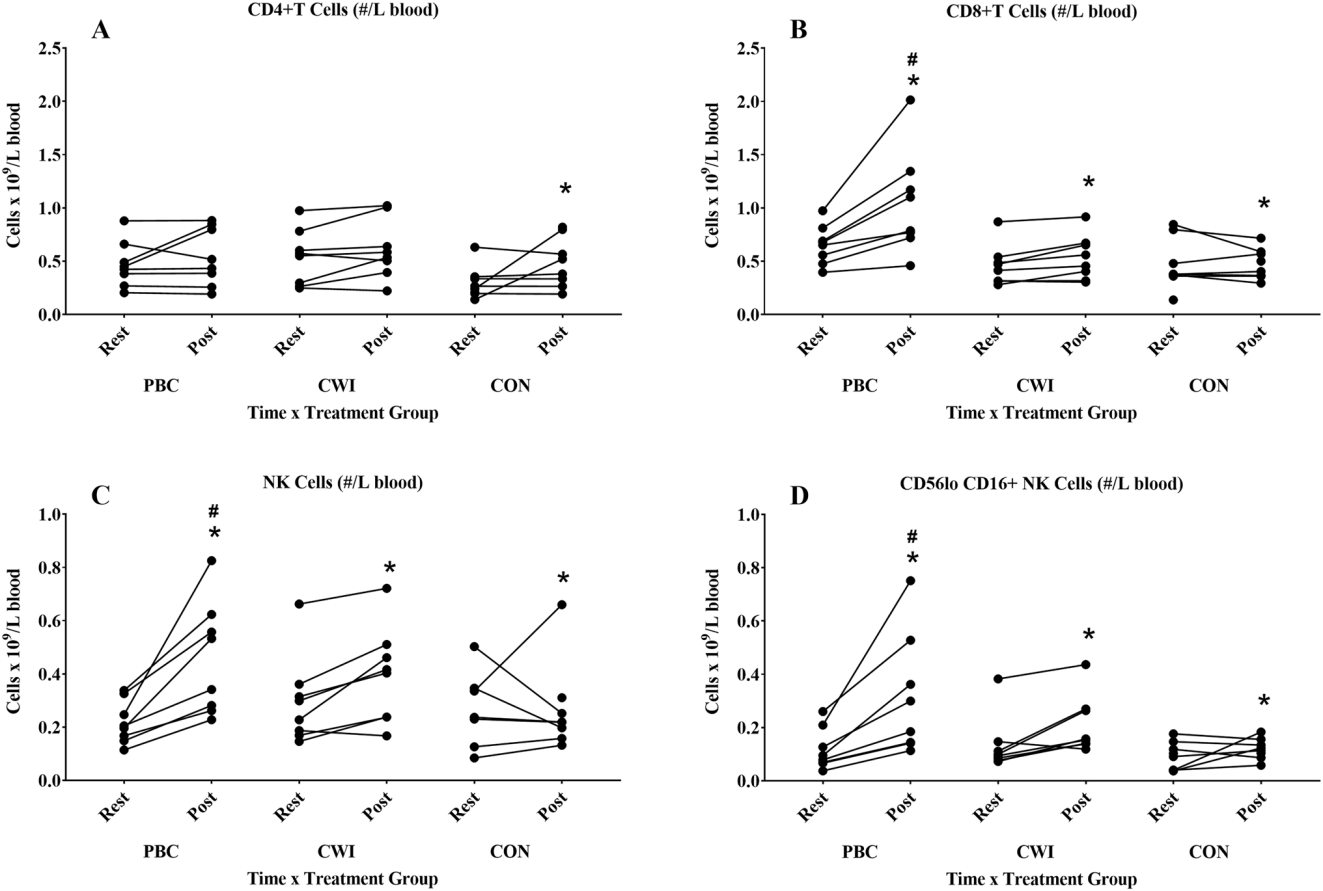
Circulating cell numbers for individual study participants. CD4^+^ T cells (**A**), CD8^+^ T cell (**B**), NK cells (**C**), and the CD56^lo^CD16^+^ NK cell subset (**D**) at 15 min of rest, and at 30 min post-exposure. *Denotes significant time effect; ^#^denotes a significant time-by-treatment effect (*p* ≤ 0.05). *PBC* partial body cryotherapy, *CWI* cold water immersion, *CON* control. Paired numbers from each participant are joined by a line

The cell concentrations of 14 individual immune populations for each group over time were assessed, to ascertain whether cryotherapy treatments lead to changes in immune cell subsets within peripheral blood mononuclear cells (PBMCs), as can be seen in Table 1. There was no significant treatment-by-time effect on quantity CD3^+^ T cells are of total immune cells in PBMCs, ‘percentage of total’ (*F* (2, 20) = 3.48, *p* = 0.05, *ƞp*^2^ = 0.26) when quantified by mass cytometry. However absolute T-cell counts were significantly different between rest and immediately post-treatment time points (*F* (1, 20) = 13.37, *p* ≤ 0.01, *ƞp*^2^ = 0.40). For the CD4^+^ T-cell subset, no treatment-by-time effect was recorded (*F* (2, 20) = 0.22, *p* = 0.81, *ƞp*^2^ = 0.02), while a significant time effect was also observed in absolute cell number (*F* (1, 20) = 5.72, *p* = 0.03, *ƞp*^2^ = 0.22) (Fig. 1A). Transient mobilisation of CD8^+^ T cells was seen, with a significant treatment-by-time effect (*F* (2, 20) = 9.92, *p* ≤ 0.01, *ƞp*^2^ = 0.50), as well as a significant time effect (*F* (1, 20) = 11.44, *p* ≤ 0.01, *ƞp*^2^ = 0.36) (Fig. 1B). Pairwise comparisons identified a significant difference in CD8^+^ cell numbers between the PBC group and the CWI group (*p* = 0.02) and CON groups (*p* = 0.01) at the post time point. This indicates that PBC treatment significantly increased the number of CD8^+^ T cells after exposure in comparison to CWI treatment.

A significant treatment-by-time interaction was observed in the number of circulating B cells (defined by CD19 + expression) (*F* (2, 20) = 4.13, *p* = 0.03, *ƞp*^2^ = 0.29), but no overall effect of time was found (*F* (1, 20) = 2.51, *p* = 0.13, *ƞp*^2^ = 0.11). Conventional monocyte populations (defined as CD14^+^CD16^−^) were not significantly different between treatments over time (*F* (2, 20) = 2.98, *p* = 0.07, *ƞp*^2^ = 0.23), nor were they altered over time (*F* (1, 20) = 0.18, *p* = 0.89, *ƞp*^2^ ≤ 0.01). A significant treatment-by-time effect was observed in CD14^+^CD16^−^ non-classical monocytes (*F* (2, 20) = 5.28, *p* = 0.01, *ƞp*^2^ = 0.35); however, pairwise comparisons indicate that this result is driven by a significant difference between the PBC and CON groups at rest (*p* = 0.01), indicating an initial subject-dependent variation in these groups. Numbers of non-classical monocytes showed no significant change over time (*F* (1, 20) = 0.05, *p* = 0.83, *ƞp*^2^ ≤ 0.01). Due to the baseline difference in non-classical monocyte populations between groups, we performed an alternative analysis comparing the delta score between baseline and post-samples and adjusting for baseline value. This approach confirmed the same pattern of findings that there was no significant change over time between groups.

The total number of NK cells exhibited a significant time effect between rest and post time points (*F* (1, 20) = 13.54, *p* ≤ 0.01, *ƞp*^2^ = 0.40). Pairwise comparisons determined that this was driven primarily through an increase in NK numbers after PBC treatment (*p* ≤ 0.01), with no significant effect detected between timepoints in either the CWI or CON groups (Fig. 1C). No significant effects were found for treatment-by-time (*F* (2, 20) = 0.63, *p* = 0.54, *ƞp*^2^ = 0.06), or time (*F* (1, 20) = 0.09, *p* = 0.76, *ƞp*^2^ = 0.01) amongst the minor CD16^−^ NK cells. However, the main CD16^+^ NK cell subpopulation (Fig. 1D) exhibited a significant treatment effect (*F* (2, 20) = 4.94, *p* = 0.02, *ƞp*^2^ = 0.33), and a difference between time points was also significant (*F* (1, 20) = 20.06, *p* ≤ 0.01, *ƞp*^2^ = 0.50). PBC was significantly different between time points (*p* ≤ 0.01), compared to no significant impact of treatments between time points for either CWI (*p* = 0.06) and CON groups (*p* = 0.48) in pairwise comparisons.

**Fig. 2:**
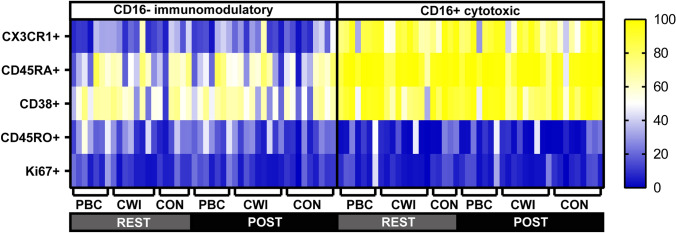
Phenotype of CD16^−^ and CD16^+^ NK cells in individual participants. Each row in the heatmap indicates the percentage of cells positive for each marker listed on the left, while each column represents an individual participant, grouped as indicated by time of sample and treatment group. *Rest* 15 min of rest, *post* immediately post-exposure, *PBC* partial body cryotherapy, *CWI* cold water immersion, *CON* control group

Given the specific increase in CD16^+^ NK cells following PBC, we sought to examine the phenotype of these cells compared to CD16^−^ NK cells across the treatment groups and timepoints. The CX3CR1 chemokine receptor, previously linked to epinephrine-mediated lymphocyte redistribution, was highly expressed on CD16^+^ NK cells (Dimitrov et al. 2010). The CD16^+^ subset was also enriched for the RA isoform of CD45 (with minimal expression of the RO isoform) and high CD38 expression. None of these phenotypes were altered across treatment (Fig. 2). Importantly Ki67, a marker of recent proliferation, was not modulated, consistent with redistribution rather than proliferation as the underlying cause of the increase in circulation of CD16^+^ NK cells following PBC treatment.

## Discussion

To our knowledge, this study is the first to investigate the mobilisation of lymphocytes after a single PBC or CWI treatment compared to a control. As hypothesised, a single cold exposure increased the circulating numbers of specific PBMC subsets, namely CD16^+^ NK cells and CD8^+^ T cells, with greater increases after PBC compared to CWI. Total NK cell counts were also found to increase after PBC, but not after CWI or CON treatments. This was likely driven by the increase in the CD16^+^ NK cell subset, which comprises the majority of circulating NK cells. Taken together, our results indicate that acute cold exposure elicits an increase in cytotoxic and tissue-homing subsets of PBMCs, with PBC resulting in a greater response.

It is well evidenced that preferential mobilisation of tissue-homing and cytotoxic cells often occurs because of an increase in epinephrine, suggesting that there is a functionally relevant response in the specific demargination of cells in response to stress (Bosch et al. 2005; Campbell et al. 2009; Dhabhar et al. 2012; Campbell and Turner 2018). Here, we show that the changes elicited by exposure to cold treatments are confined to CD16^+^ NK and CD8^+^ T-cell subsets and are increased in response to lower temperatures. Characterisation of CD16^−^ and CD16^+^ NK cells within each blood sample provided insights into the origin of the changes in cell numbers. The phenotypes of the two NK cell subsets were stable across all treatment groups and times, indicating that the greater number of circulating CD16^+^ NK after PBC treatment was unlikely to have been due to an alteration in phenotype or an increase in cell division (marked by expression of the Ki67 antigen). More likely, the change may be due to an increase in catecholamines, in line with the higher HR that we observed in response to PBC (Horowitz et al. 2013; Bigler et al. 2015). This conclusion is supported by the study of Leppaluoto et al., who demonstrated that PBC induces a significant catecholamine response (Leppaluoto et al. 2008).

Hypothermic stress as a result of exposure to cold environments and cold water has been shown to increase circulating catecholamine concentration and subsequently influence lymphocyte mobilisation (Jansky et al. 1996; Castellani et al. 2002). Our results found that PBC stimulated a greater change in numbers of CD16^+^ NK cells and CD8^+^ T cells in comparison to CWI, suggesting a larger catecholamine response after PBC could result in a more-pronounced mobilisation of lymphocytes than CWI. This response is similar to that found after exposure to other stressful stimuli, such as exercise, injury, or psychological stressors (Castellani et al. 2002; Dhabhar 2002; Dhabhar et al. 2012). CD16^+^ NK cells in this study were observed to increase by 167% after PBC, 58% after CWI, and 29% after CON. Exercise, as a form of physiological stress, will also result in an intensity-dependent immediate increase in numbers of circulating lymphocytes (Campbell and Turner 2018). A 935% increase in the number of CD16^+^ NK cells in circulation was observed during a high-intensity 20 min cycling effort at 85% of athlete’s maximum effort (Campbell et al. 2009). Similar trends in lymphocyte mobilisation have been reported with a 206% increase in CD16^+^ NK cells observed after a 4 min public speaking task (Bosch et al. 2005). It may be assumed, from this evidence, that while there was an impact of PBC and CWI on CD16^+^ NK and CD8^+^ T-cell mobilisation, the effect is not as pronounced as after a high-intensity bout of exercise, but is similar in magnitude to the stress of a public speaking task. With these cells involved in reparation of cell damage and various other immune functions, a transient increase in circulating numbers using cold therapies may be beneficial in moderating modest immediate challenges to the immune system and requires further investigation for appropriate application.

No alterations were observed in the number of circulating conventional monocytes with any treatment group. Between rest and post-treatment, an increase in B-cell counts was recorded in the CON group, but not in either PBC or CWI groups. This result, while statistically significant, is likely a result of intrasubject variation between time points. Differences in non-classical monocyte numbers between PBC and CON were found only at rest and are therefore likely due to inter-subject variation between these treatment groups at baseline.

While the increase in CD16^+^ NK and CD8^+^ T cells after a single 3 min exposure to PBC suggests an acute stress response, differences other than temperature and limitations in this study should also be acknowledged. There were unavoidable variations in posture during exposure to the various treatments, due to the constraints in equipment design. Thus, PBC treatment was conducted standing in the chamber, the CWI treatment involved participants sitting with legs extended on the floor, and the CON group were seated in a chair with feet on the floor. These postural differences may impact HR and BP responses and should be considered in future study designs. The testing was conducted in a high-performance sports science facility, unfamiliar to participants, and involved exposure to novel recovery treatments which may have increased psychological anxieties and influenced leukocyte populations within participants. A period of familiarisation with both the facility and treatment exposures, followed by a suitable wash out period, may be beneficial in mitigating any effect of the novelty of treatments. A limitation of this study is the small sample size for PBMC analysis, and the recruitment of males only. These preliminary findings hold a lot of promise and along with careful implementation of experimental controls to enable larger comprehensive immunophenotyping studies, we look forward to investigating the impact of this treatment with larger sample sizes including women in future investigations. These future studies could utilise a cross-over design strategy to overcome group inter-participant variation we encountered in this pilot study. Additional, to ascertain dynamics multiple time points (in the hours post-treatment) could be included to assess tissue immune surveillance.

## Conclusion

The increase in preferential redistribution of specific PBMC subsets involved in an acute stress response observed after PBC may have as yet unexplored applications. A larger number of circulating immune cells, particularly those with specific tissue-homing and cytotoxic functions, may be immunoenhancing and increase protection against antigens (Viswanathan and Dhabhar 2005; Rosenberger et al. 2009; Dhabhar et al. 2012; Dhabhar 2014). In terms of recovery from injury or insult to muscle tissue, animal studies have established that when these cells disappear from circulation, they migrate to areas of damage which may expedite recovery processes involving these migrating cells (Dhabhar 2009; Dhabhar et al. 2012; Campbell and Turner 2018). In this context, application of PBC as a therapy may be considered outside of sports medicine and recovery from EIMD. Ongoing investigations should attempt to understand the impact of this transient lymphocytosis after cold exposure, with relevance to various potential therapeutic applications.

### Practical implications

- Acute 3 min exposure to PBC elicits a greater lymphocytosis response than CWI
- The acute lymphocytosis after PBC exposure is comparable to a public speaking test, but less stressful than a bout of vigorous exercise
- The transient increase is specific to CD8^+^ T cells and CD16^+^ NK cells, consistent with a process that mobilises immune cells with specific functional properties related to tissue healing and repair.
