# Correction to: Partial body cryotherapy exposure drives acute redistribution of circulating lymphocytes: preliminary findings

**DOI:** 10.1007/s00421-022-05100-4

**Published:** 2022-12-05

**Authors:** Catriona L. Rose, Helen McGuire, Kenneth Graham, Jason Siegler, Barbara Fazekas de St Groth, Corinne Caillaud, Kate M. Edwards

**Affiliations:** 1 Faculty of Medicine and Health, Sydney and School of Health Sciences, Discipline of Exercise and Sport Science, The University of Sydney, Charles Perkins Centre, Camperdown, Sydney, NSW 2006 Australia; 2 Faculty of Medicine and Health, Sydney and School of Medical Sciences, Discipline of Pathology and Ramaciotti Facility for Human Systems Biology, The University of Sydney, Sydney, NSW Australia; 3 Applied Research Programme, New South Wales Institute of Sport, Sydney, Australia; 4 Sport and Exercise Science, School of Science and Health, University of Western Sydney, Sydney, NSW Australia

Correction to: European Journal of Applied Physiology 10.1007/s00421-022-05058-3.

The original version of this article unfortunately contained a mistake. The given name and family names of five author were incorrectly structured.

The correct given name and family names should be:

Helen McGuire.

Kenneth Graham.

Jason Siegler.

Corinne Caillaud.

Kate M. Edwards.

The original article has been corrected.

